# Beyond compliance: Good citizenship during the COVID‐19 pandemic

**DOI:** 10.1111/tran.12587

**Published:** 2022-11-11

**Authors:** Nick Clarke, Clive Barnett

**Affiliations:** ^1^ Geography and Environmental Science University of Southampton Southampton UK; ^2^ School of Geography University of Exeter Exeter UK

**Keywords:** citizenship, COVID‐19 pandemic, Mass Observation, responsibility, risk, United Kingdom

## Abstract

In the UK, discussion of good citizenship during the COVID‐19 pandemic largely focused on compliance and non‐compliance with government rules. In this paper, we offer an alternative point of focus. Pandemic governance proceeded not only through rules/morality, but also through freedom/ethics. Good citizenship, therefore, involved practical reasoning in response to situations. We demonstrate this using diaries and other forms of writing collected by Mass Observation during the first six months of the pandemic. Responses to government rules and guidance varied by situation. Many people found governance through freedom/ethics confusing and burdensome. Faced with ethical dilemmas, they managed risks and responsibilities by deliberating, weighing justifications, and sometimes falling back on rules of thumb or heuristics. Discussion of good citizenship during future emergencies would benefit from a greater focus on situations, dilemmas, and justifications.

## Short Abstract

Good citizenship during the COVID‐19 pandemic involved not only compliance with rules but also practical reasoning in response to situations. We demonstrate this using diaries and other forms of writing collected by Mass Observation.

## 1 GEOGRAPHIES OF CITIZENSHIP AND RESPONSIBILITY

During the COVID‐19 pandemic, there was much discussion of good (and bad) citizenship. In the UK, until a significant proportion of the population was vaccinated, much of this discussion focused on (non‐)compliance with government rules concerning geographical behaviour (e.g., Reicher & Stott, 2020; Webster et al., 2020; Wright et al., 2021) – rules requiring people to stay at home, meet others outside, keep two metres apart, wear face coverings in certain places, and so on. One prominent view in this discussion was developed by psychologists and behavioural scientists, who argued that: rates of compliance were generally high (Fancourt & Steptoe, 2020); examples of non‐compliance were explained less by psychological factors such as panic or fatigue, and more by systematic factors such as just‐in‐time supply chains or lack of government support (Drury et al., 2020); and the preoccupation of government ministers and journalists with examples of non‐compliance led to policy failures, including delayed lockdowns and public health messaging that set norms of non‐compliance for previously compliant people to follow (Reicher & Drury, 2021).

In this paper, we offer a complementary view developed from three starting points: an observation of how pandemic governance in the UK varied over time; a reading of the ‘geographies of citizenship’ literature; and analysis of diaries and directive responses collected by Mass Observation during the first six months of the pandemic.

In the UK, governance of the pandemic developed over time. An initial focus on rules in spring 2020 – the UK's first lockdown – was replaced in summer 2020 by a focus on ‘freedom’, ‘common sense’, and ‘personal responsibility’. After this, during the rest of 2020 and for much of 2021, governance of the pandemic fluctuated between periods of lockdown and ‘relaxation’, depending on infection rates and other considerations. We might say that governance of the pandemic worked, or tried to work, initially through morality (rules), but later through ethics (freedom, choice, responsibility). And we might say this latter mode of governance caused problems for some commentators. It demanded a focus beyond compliance and non‐compliance. It demanded an imagination of citizenship as not only formal and liberal, but also substantive, communitarian, and radical democratic (Delanty, 2000); as not only duty‐based and elitist, but also engaged (Dalton, 2009); as not only traditional and conventional, but also critical (Norris, 1999). It demanded that ‘common sense’ be taken seriously – as the repertoire of ways people think, feel, and do when confronted by problems – and not just dismissed as government or party ideology (e.g., Earle, 2021; West, 2020). It demanded that ‘personal responsibility’ be taken seriously and not just relegated to one of many ‘unknowns’ driving uncertainty and variation in epidemiological models (e.g., Thompson et al., 2020).

Good citizenship during the pandemic, then, involved not only compliance with rules, but also the exercise of freedom, choice, and responsibility. The literature on geographies of citizenship can help us to approach such a more broadly conceived good citizenship. Geographers have been studying the management of risk, responsibility, and geographical behaviour by engaged, critical citizens operating in contexts of relative freedom for at least two decades. They have done so in response to so‐called ‘real‐world problems’ like international development, globalisation, climate change, and multiculturalism. And they have done so in response to at least two theoretical developments: Beck's risk society thesis, in which modernity produces risks and associated management of risks and responsibilities by various actors, including individuals (Beck, 1992); and Foucauldian governmentality studies, in which one definition of liberalism is government by risk management, and one definition of advanced liberalism is government by individual responsibility, including responsibility for risk management (Dean, 1999; Rose, 1999).

The geographies of citizenship literature covers many themes. One prominent theme is the development of citizenship in response to globalisation, transnationalism, European integration, and decentralisation (Barnett et al., 2011; Desforges, 2004; Ehrkamp & Leitner, 2006; Hörschelmann & El Refaie, 2014; Lough & McBridge, 2014; Mäkinen, 2021). These processes have encouraged a geographical view of citizenship as multi‐dimensional, multi‐sited, and multi‐scaled. Another prominent theme is the relationship between advanced liberalism – or, more commonly in the literature, ‘neoliberalism’ – and various dimensions of citizenship (Ghose & Pettygrove, 2014; Kearns, 1995; Leitner & Strunk, 2014; Mitchell, 2005; Painter & Philo, 1995; Swyngedouw, 1996; Teo, 2015). Neoliberalism is thought to involve: privileging the rights of individual, autonomous, private citizens; downloading responsibilities for welfare to individual citizens; limiting opportunities for meaningful participation by citizens; granting members of different groups different rights (graduated citizenship) in exchange for different behaviours (conditional citizenship); and struggles for alternative rights, duties, and spaces for participation and belonging. The COVID‐19 pandemic is thought to have exposed and exacerbated some of these neoliberal citizenships. For example, Schliehe et al. (2022) argue that British prisons used the emergency of the pandemic as an opportunity to retrench and rework the carceral, extending or introducing new population management strategies, micro‐spatial partitioning, extensive isolation, enforced segregation, and reduced association time.

For the purposes of the present paper, two insights from the geographies of citizenship literature are particularly important. First, people are geographical beings whose moral concerns emerge from their geographical actions and awareness (Sack, 1997). Therefore, ethics, politics, responsibility, democratic agency, and good citizenship are all contextual and relational. They emerge from situations or locales or lifeworlds that provide resources and opportunities for practices, encounters, interactions, and experiences (Askins, 2016; Barnett, 2017; Clarke et al., 2017; Jackson & Valentine, 2014; Massey, 2004; Popke, 2009; Smith, 1999). ‘Actually existing citizenship’ (Spinney et al., 2015), then, is ‘ordinary’ (Staeheli et al., 2012): framed by laws and policies, but negotiated and performed in response to norms, values, and everyday contexts. Second, once responsibility emerges from situations, people argue about it (Hinchliffe, 1996). They do ‘lay normativity’ (Sayer, 2004). They treat norms as normative, and reason in response to them – using practical reasoning skills to learn norms, but also to interpret and make judgements in relation to them (Barnett, 2015). People act with discretion, expect to be judged, and give accounts of their actions populated by reasons and justifications (Barnett, 2015; see also Barnett, 2012, 2014). They carry multiple prior commitments and engage with responsibility talk – especially demands that people ‘take responsibility’ – critically (Barnett et al., 2011). Therefore, if policy‐makers wish to encourage good citizenship in the form of risk and responsibility management by citizens, they need to provide information (allowing people to make informed decisions), practical support (allowing people to act on those decisions and put them into practice), and, crucially, justifications people can use in the arguments that almost inevitably arise during responsibility talk (Barnett et al., 2011; see also Barnett, 2010). It should be added here that geographers have developed these insights alongside scholars from allied disciplines attuned to contexts, situations, relations, interactions, norms, and values. Mary Douglas from anthropology is one example. For Douglas (1994), risk and responsibility are connected. Risk decisions are intersubjective, social, and taken in consultation with family, friends, and colleagues. They are not made in isolation, but in comparison to multiple other risks and cared about objectives and goals. They are moral decisions, made in relation to values.

In this paper, we consider good citizenship during the pandemic from a geographical perspective, or at least a contextual, situational, relational perspective. We argue that not only has there been too much emphasis on non‐compliance at the expense of compliance (the concern of the psychologists introduced previously), but also there has been too much emphasis on compliance at the expense of other aspects of good citizenship. We focus on the following questions. What did people do when asked to use common sense, to act responsibly, and to manage risk by adjusting their geographical behaviour during the pandemic? How did people interpret such demands? How did they act on them? How did they justify their responses? How was all this affected by situations made up of resources, opportunities, practices, relations, norms? And how was all this governance through freedom/ethics experienced? We address these questions drawing on volunteer writing for Mass Observation, which provides our focus on the UK in spring and summer 2020.

## 2 MASS OBSERVATION'S COVID‐19 COLLECTIONS

Mass Observation (MO) was originally founded in 1937. Throughout its history (see Hinton, 2013; Hubble, 2010), it has combined a dual focus on ordinary life – what people do, think, and feel on a daily basis – and popular responses to extraordinary events, e.g., the Coronation of George VI, the Second World War, the period of high inflation at the beginning of the 1980s, and, most recently, the COVID‐19 pandemic. Today, MO consists of: the Mass Observation Archive (MOA), established in 1975; the Mass Observation Project, established in 1981 by the MOA, which sends ‘directives’ three times a year to a panel of roughly 500 volunteer writers from across the UK, asking panellists to write about particular topics for the Archive; and the 12 May project, established in 2010 by the MOA, which publishes annual calls asking anyone resident in the UK to keep a ‘day diary’ on 12 May for the Archive (inspired by the original MO's first book, *May the Twelfth* – Jennings & Madge, 1937).

In 2020, MO asked about life during the COVID‐19 pandemic in its regular spring and summer directives, a special directive sent on 17 March at the start of the UK's first lockdown, and the call for 12 May day diaries (see Appendix S1: Section 1). The writing sent back to MO in response to these calls, along with responses to other calls made during 2021, makes up MO's COVID‐19 collections. In this paper, we focus on that part of the collections covering the period of the UK's first lockdown (March to May 2020) and the ensuing period of opening up, before restrictions were brought back for some regions in July, nationally in September, and formally as the UK's second lockdown in November 2020.

What does this collection of archived writing – diaries, letters, and other forms – tell us about life in the UK during the COVID‐19 pandemic? More broadly, how should MO sources be interpreted? There is a mature literature on this latter question spanning the social sciences and humanities. One thing to learn from this literature is that writing for MO is dialogic and subjective. It is part of a dialogue with MO (Bloome et al., 1993; Salter, 2010), with panellists best thought of not as respondents but as *cor*respondents (Sheridan et al., 2000) who exchange writing with MO as part of an ongoing conversation with a purpose, which is to document everyday life for an imagined audience of current and future researchers (Kramer, 2014). It is also subjective writing in the service of identity construction (Nettleton & Uprichard, 2011) and especially the positioning of panellists in relation to MO and other authorities and institutions (Sheridan et al., 2000). Writing for MO, then, provides evidence of top‐down and bottom‐up processes. In the diaries and letters of panellists, we see their cultural worlds – their worlds of discourse – and how they construct from these worlds their own distinct selfhoods (Hinton, 2010). We see evidence of standards and codes, but also how people receive such standards and codes, their dis/comfort with them, and what people do with them (Langhamer, 2016). We see the sociological constructs panellists use as resources, but also their lay articulations of those constructs – their practices of selection, interpretation, appropriation, incorporation, and contextualisation (Wilson‐Kovacs, 2014).

From the above paragraph, we conclude that MO sources are particularly well suited to researching how people practise their lives in relation to audiences, authorities, institutions, discourses, standards, and codes, and so are particularly well suited to our purposes in this paper. Furthermore, one particular part of MO's COVID‐19 collections is especially well suited to our focus on governance through freedom/ethics: the 12 May day diaries. These were kept by just over 5000 people across the UK on 12 May 2020, just two days after the Prime Minister announced the end of the first lockdown. As such, they capture an important moment when governing the pandemic through rules/morality (e.g., the ‘instruction’ to ‘stay home’) was supplemented and, for a time, largely replaced by recommendations that people ‘stay alert’ and use ‘common sense’ to manage their own risks and responsibilities. In the 12 May diaries, we see people beginning to work through the implications of this change. In these and other materials from subsequent weeks, we see people working out how to manage their own risks and responsibilities, how to reason about and justify their own hygiene and associated practices, and how to resolve the multiple ethical dilemmas thrown up by the novel situations in which they find themselves.

If these are the advantages of MO's COVID‐19 collections – that writing for MO demonstrates how people respond to demands and situations, and materials from the period following 10 May 2020 capture the shift in governmentality from rules/morality to freedom/ethics – then the most commonly discussed disadvantage of MO, especially the directive responses, is the social constitution of MO's panel of volunteer writers. This panel has never been statistically representative of the UK population as a whole. In recent decades, it has been skewed a little towards women, older people, the middle classes, and England (Hinton, 2013). Certain groups and experiences are largely missed by the panel, e.g., the experiences of prisoners ‘locked down under lockdown’ during the COVID‐19 pandemic (on which see Schliehe et al., 2022). What can be said *for* the panel is that good citizens are well‐represented. By definition, panellists are volunteers (who volunteer for MO's social history project). Hinton (2010) found them to be ‘active citizens’: participants in society as members of families and friendship networks, and as consumers and workers; people on a quest for meaning and purpose in their lives beyond the mundane satisfactions of everyday life; and particularly reflective people who provide access through their writing to the cultural worlds inhabited by themselves and others. Manning (2018) found them to be citizens who generally vote and engage with representative democracy in other respects (e.g., keeping themselves informed regarding political issues and options). Lindsey and Mohan (2019) found many panellists to be volunteers not only for MO, but also for other civil society organisations and projects (of the kind prominent during the COVID‐19 pandemic, e.g., mutual aid organisations and projects – on which see Mould et al., 2022). All this is consistent with Pattie et al.'s (2004) findings on citizenship in general: that people who volunteer for one organisation or project (e.g., MO) tend to practice good citizenship in other ways too – by volunteering for other organisations and projects, and by participating in politics (both formally, e.g., voting, and informally, e.g., signing petitions).

Again, this focus on good citizens is well‐suited to our purposes in this paper. In order to research good citizenship during the pandemic, we are interested in precisely these citizens. Furthermore, it is possible to sample within the panel – or the writers, in the case of the 12 May diaries – which we did for each of the collections. Following Clarke et al. (2018), we sampled 60 writers per collection, which was enough to achieve descriptive saturation and also, for the most part, to fill quotas for gender, age group, region, and occupational classification (see Appendix S1: Section 2). In total, we read 180 pieces of writing from 60 day diarists and 60 panellists (two directive responses per panellist). Together, these made up almost 800 sides of A4 (typed and single‐spaced). We read this material for stories of risk and responsibility management, working independently at first, then comparing notes, then, where agreement could be reached on interpretations – a version of intercoder reliability – sorting the material accordingly.

We return to the question of representativeness in the concluding section of this paper, where we situate our findings from the MO data in relation to findings from available survey data, before clarifying what the paper adds to existing portraits of good citizenship during the COVID‐19 pandemic. For now, having addressed the sampling, or statistical, or input problem of representativeness (described in the previous paragraph), let us briefly attend to the output, literary, aesthetic problem of representation. This has often been a point of debate between users of MO sources, with social scientists more focused on the problem of representativeness and those in the humanities more focused on the problem of representation. In this paper, we seek to address both problems (so far as that is possible within the word limit). Regarding the latter problem – the aesthetic problem of representation – we take lessons from histories of the original MO (Highmore, 2002; Hubble, 2010; Jardine, 2018; Marcus, 2001). The founders of MO in the late 1930s were influenced by Surrealism and sought to represent everyday life using aesthetic techniques from surrealist poetry, painting, film, and theatre. We seek the same in the rest of this paper: depicting pandemic life in the UK using panoramas and close‐ups, typical and atypical cases, juxtapositions of different and partial perspectives; providing a composition, a collage or montage, appropriate to British society (a heterogeneous ‘totality of fragments’ – Highmore, 2002).

## 3 STORIES OF RISK AND RESPONSIBILITY MANAGEMENT

### 3.1 From ‘stay home’ to ‘stay alert’

Many of the 12 May diaries and the longer diaries kept by panellists in response to MO's special directive – some for a period of six months or more – capture the moment on 10 May when government messaging shifted from ‘Stay home, protect the NHS, save lives’ to ‘Stay alert, control the virus, save lives’. Diarists were generally confused by the shift and viewed the new rules and guidance as less clear and more open to interpretation. They ‘found this change in slogan confusing’ (MT 2020 37) and ‘very muddled and unclear’ (MT 2020 132). They were ‘utterly baffled’ (K7050) by the new guidelines, which they found ‘very confusing’ (M3231).

Many diarists now realised they had to calculate, manage, and justify their own risks and responsibilities. An air traffic planner in his 50s from Hampshire (D4736) found the Prime Minister's press conference on 10 May to be ‘vague and open’. There was ‘not enough detail’. His family were left ‘scratching our heads wondering what this new “stay alert” phase means. Will [wife] be able to go swimming? Could I venture up to Heathrow [to go plane spotting]? Can we go and visit [daughters]?’ There were ‘so many questions’.

How would people answer these questions? Some panellists picked up from government messaging that ‘common sense’ should be used. They were not impressed. A retired teacher in his 60s from Somerset (MT 2020 119) wrote: ‘The government's communications have become increasingly confused and confusing’. With due scepticism, he continued: ‘The PM who boasted (pre‐becoming infected with COVID‐19 and nearly losing his life) of shaking hands with everybody despite scientific warnings that we should not be shaking hands has advised the British public to use “good solid British common sense” in interpreting the new lockdown rules’. Similarly, a woman in her 70s from Warwickshire (T6654) described ‘the whole thing’ as ‘totally confused and muddled – leading to a dangerous situation where eventually the public was just told to “use your common sense”’. Like the previous diarist, she regarded this promotion of common sense with scepticism. If such a thing as common sense exists, presumably it describes what people perceive, think, feel, and do when confronted by problems. In the rest of this paper, we describe the content of such common sense for the period following the 10 May press conference. We finish the current set of points by emphasising that many panellists did not welcome their new freedoms around this time. In these freedoms, they saw responsibilities. They were ‘angry and frustrated’ by the ‘inconsistent and confusing’ guidance (MT 2020 250). They were ‘fed up of all the announcements and lack of clarity’, and ‘confused about the new message’ (MT 2020 542). They wore this new demand to act responsibly like a heavy burden.

### 3.2 Situations for minimising risk

How this demand was met, of course, varied by situation. Writers who saw themselves as vulnerable – often because ministers, civil servants, scientists, and/or journalists had seen them as vulnerable – took extra care to minimise risk. A graphic designer in his 20s from Suffolk (MT 2020 221) wrote: ‘I am deemed vulnerable, as I have an underlying health condition. As a household, we have needed to be extra cautious, with our travel significantly reduced and taking the initiative with additional precautions such as wearing gloves and masks in shops – weeks before it became recommended’. He also took walks early in the morning, ‘when there are barely any other people out walking, so the risk of encounters is reduced’. His partner did all their ‘in‐store shopping, to protect me from coming into contact with people’. This couple were not simply adhering to rules and guidance. They were ‘being extra cautious’, ‘taking the initiative’, taking ‘additional precautions’, and doing so ‘before it became recommended’. They were taking risks appropriate to what they understood of their own particular situation. And they were allocating responsibilities appropriately too (with his partner, deemed less vulnerable, taking responsibility for shopping trips).

There were many other examples of ‘additional precautions’ taken by ‘vulnerable’ writers, including the couple in their 70s with ‘health issues’ who were ‘particularly careful’, exercising ‘early in the morning to avoid everyone’, visiting the Post Office at ‘what I thought would be a quiet time’, purchasing the newspaper ‘wearing gloves’, and putting its outer pages ‘in the microwave to disinfect it’ (P1796). But let us turn to another group: key workers. This was another new social identity inhabited by some. And again, it carried its own set of risks and responsibilities to be managed. A retired nurse in her 50s from Cambridgeshire (MT 2020 284) wrote of her husband: ‘he is a “key worker”’. He wears ‘a cloth face covering’ at work. He ‘immediately washes his hands when he arrives home’. A clinical pharmacist in her 40s from Cheltenham (MT 2020 479) described her own post‐work routine as follows: ‘After work each day I need to put my clothes in a bag and leave them for three days before washing them and then shower to reduce the chances of COVID infection. I shower using plenty of shower gel and shampoo and then dress in clean clothes and join my children to catch up on their day’. There were few correspondents working from home during this period who felt the need – the responsibility – for such a laborious post‐work routine before seeing their children.

Nevertheless, many panellists – as good citizens writing for MO – did seek to reduce risk to themselves and others during spring and summer 2020. They timed and located their daily walks for quiet periods and places, getting up ‘at 6 am to avoid the crowds’ (M6737) or walking ‘late in the evening in order to avoid lots of people’ (W7312). They cleaned themselves, their clothes, and their homes more regularly. A retired university administrator in her 60s from Oxfordshire (M6749) described her ‘dry and cracked’ hands, before writing: ‘I have always hated housework and cleaning but push myself into an hour's cleaning and sanitising every day whether I feel like it or not’. She continued: ‘And who would have thought that we would be washing the shopping?’ She was not alone in going to these lengths. Consider this routine of a retired writer in his 70s from Lancashire (W3176) on returning from ‘a shopping expedition’: ‘Hand washing immediately’, followed by ‘Handrails, door handles, wheelie bin grips, and even the steering wheel of the car’, which all ‘receive attention, courtesy of cleaning with antiseptic wipes’. These risk‐conscious panellists, often older in age (and so vulnerable by some definitions), also avoided certain places perceived to be risky – especially public transport. A woman in her 80s from Essex (B7306) explained that she would not be seeing her family because she was ‘dependent on public transport’ and ‘apprehensive about the dangers of infection while travelling’. Another woman in her 80s, this one a retired shop worker from Yorkshire (J1890), kept ‘putting off’ visiting the dentist and optician ‘because I don't want to go on a bus’. A woman in her 60s from Fife (H6675) gave the following justification for missing her granddaughter's school show: ‘want to avoid trains’. She went on to describe risk management during this period as ‘an exhausting game, having to think about every move you make, and how to do things’.

The game was exhausting partly because it involved extra washing and cleaning, but partly because it was difficult to know quite when to stop, where to draw the line, and what was appropriate or excessive. ‘Is opening a gate with a stick being over‐cautious?’, as one panellist put it (H6004). In the same paragraph, this retired civil servant in his 60s from Yorkshire wrote: ‘Oddly, given that restrictions are being eased, I feel more rather than less glum. One factor is uncertainty about the right level of caution to be exercised’. This was one responsibility allocated to citizens by the decision to relax lockdown rules on 10 May: the responsibility to decide how much caution to exercise. The other side of this was a related but different responsibility: to decide how much risk to take (by leaving home, taking trips, meeting people, and so on).

### 3.3 Justifications for taking risks

In the writing of MO correspondents, risk‐taking was justified as ‘essential’ or ‘acceptable’ in multiple terms: competing risks, such as to the wellbeing of selves (at risk of reduced mental wellbeing if denied a trip or meeting); competing responsibilities, such as for the wellbeing of others with care needs; public health messaging about levels of risk and available mitigation measures (social distancing, ventilation, face coverings etc.); infection rates (nationally, but especially locally); what feels right (quiet, clean, safe); and what is practical, given the time and effort involved, and the potential endlessness and impossibility of risk management.

Much of this can be illustrated by a close‐up of an air traffic planner in his 50s from Hampshire (D4736). Like other dutiful panellists, he sought to reduce risk where possible. He cancelled a visit to a friend because ‘her son is reporting a cough and temperature’, writing: ‘I decided we should not do that […] I think the risk of staying at [the friend's] is too great’. He took packed lunches to the office ‘to avoid the canteen’. He went shopping ‘at midday, thinking it might be a quieter time’. Walking the dog, he avoided the back path: ‘a bit too busy for my liking’. Like many panellists, he also worried about how much caution was necessary or sufficient. He wrote: ‘We have three parcels sitting at home waiting to go to the post office. Can't decide if this is a necessary journey/risk to take’. Later, after a trip to the supermarket, he asked: ‘what do I do with my trolley?’ (he used ‘gloves kept in the car to hold it’). Shopping was described as ‘a minefield’. He found it ‘hard to relax’. The virus ‘could be on anything, anywhere’. On arriving home, the questions kept coming: ‘Washed hands thoroughly, and face, and hands again. Blow nose. Rinse and repeat. Trouble is you've touched products which the checkout lady has too, then I come in and wash hands, put things away, touching handles in the kitchen. Where does it end?’

Despite all the worry and caution displayed in the previous paragraph, this panellist did take risks during the pandemic, justifying them in his diary, including some episodes of non‐compliance with government rules and guidance. He argued that risk management has to be ‘practicable’. When shopping, ‘Isolating/distancing is just a nightmare, so difficult to actually do’. Yet shopping must be done. He argued that rules could be broken, such as the limit of one walk per day during the first lockdown, so long as their spirit was met: ‘After tea took [dog] a quick twice around the little wood behind us to tire and settle her […] It's OK, met no one, and that's the whole point isn't it?’ He argued that infection rates were relevant to the risk‐management decisions of individuals. After non‐essential shops were reopened at the end of the first lockdown, he wrote: ‘I want to see how it goes, see if there is a spike or second wave before I relax any of the things I'm doing’. Later in the summer, when some of the rules had been relaxed, he argued that risks could be taken if mitigated following the latest advice. ‘We ended up with a bit of a little garden party this afternoon’, he confessed. They had invited some friends round. Then some other friends had turned up. In the diary, he reassures himself (and us): ‘Oh well – as I understand it, it is being indoors and touching surfaces that are the real transmission danger spots, so open air meeting is relatively safe I suppose’. Finally, and perhaps most importantly, he argued that care of loved ones, or those for whom one feels responsible, took precedence over government rules and guidance. This included care for his neighbour, whose computer he fixed, ‘considering [the visit] essential care for the elderly’. And it included care for his daughter, living alone, except for some health problems. At one point, he wrote: ‘We've decided if we have to go [and get her], it's legit travel as it's a medical emergency’. Later, with his wife, he discussed ‘bringing her home as a health need for recuperation and hugs’, asserting that ‘acute need for some looking after takes precedence in the circumstances’. Towards the end of the diary, he wrote: ‘In a way, we are breaking restrictions by having [both daughters] back in our family bubble but returning them next week. We think it's an acceptable and calculated risk’.

### 3.4 Ethical dilemmas

Good citizenship during the pandemic involved not only compliance with government rules, but also calculating risks and justifying caution or risk‐taking. Indeed, it involved practices of calculation and justification in relation to risk, but also responsibility. We have seen that such calculations could be made, with justifications provided, but ethical dilemmas were also frequently confronted, leaving people unsure how to proceed. MO correspondents wondered how to balance the demands of government – ‘stay home’, ‘protect the NHS’, ‘stay alert’, ‘control the virus’ – against other demands, rights, and goods, for which social distancing and related hygiene measures were perhaps less appropriate responses. There were the ‘vulnerable’ panellists, for whom staying at home and accepting help with tasks like shopping threatened a virtue they valued in themselves: independence. ‘For 90 years I have been independent and not been a nuisance to my family’, wrote a retired shop worker from Essex (H260). ‘We have always been incredibly independent and we did not always like to ask for favours’, wrote a student support worker from Derbyshire (W7312) – younger than the previous panellist, but shielding with her vulnerable husband. There were the panellists who valued politeness and reciprocity, and wished to respond positively to invites and offers, while worrying about the risks involved. A woman in her 60s from Fife (H6675) confessed: ‘[A friend] ran out to invite us in to view her new kitchen: I went in reluctantly and briefly; not sure I should have’. A writer in her 40s from North Ayrshire (M5645) ‘felt that’ she ‘had to go to a quiz’ organised by her neighbour because that neighbour ‘gave me a care package’ earlier in the week, ‘saying they thought I was struggling, and that they were there for me’. At the quiz, someone ‘insisted that I have a glass of pink gin, she put a tin on our table […] I felt I had to accept it to show that she was taking care of me’.

In the novel situations of the pandemic, people were asked to comply with rules and guidance, but also to compare and sort numerous demands, rights, and goods. To illustrate more of this, we provide another close‐up of the air traffic planner in his 50s from Hampshire (D4736). Ethical dilemmas appear frequently in his diary. An early one is the dilemma of social distancing (stay home, protect the NHS) versus providing care to his vulnerable neighbour. He asked himself: ‘Wonder if I should be “meeting up” with [neighbour] with the ever tighter restrictions. Know she gets depression sometimes and anxiety’. Later, a similar dilemma concerned providing care to his friends. ‘I suggested we have [friends] round for curry this evening’, he wrote. ‘I've been meaning to do this for ages to “check in” how they're doing […] to support them. Was it me or [friend] who suggested it and I enthusiastically agreed? And then I wonder if this is the right thing as both our wives are shielding’. He also wondered if it was the right thing for his wife to visit the coast to swim. Lockdown rules were being relaxed by this point in the diary, but people were still meant to be avoiding unnecessary travel. He wrote:

With the easing of lockdown, we have been figuring out what that means for us, particularly [wife's] swimming. Decided going to [regular swimming spot] would be OK. It's 40 miles, 50 minutes, there is not really anywhere closer that's ideal and it's where the group she is with all swim […] It does make a difference to [wife], she's been feeling more aches and pains and seizing up from not having this usual exercise and the mental stimulus side of it is so beneficial to her.

Travel in this case was judged to be necessary or at least defendable in terms of exercise and mental stimulus. The same judgement was not made, at least initially, regarding his own preferred activity of plane spotting. He continued: ‘My dilemma for exercise or “mental stimulus” is do I go spotting?’ He checked the ‘Government website’ for advice, concluding that it would be ‘technically OK’. But he also checked the ‘aviation forums’, where opinion was ‘split’. He proceeded by asking himself questions. ‘If I am careful and sensible as we are being it'd be OK? […] Should I go or can I wait?’ In the diary, plane spotting is described as ‘a trivial need, superfluous. Just a tick in a book’. But it is also described as ‘what I do’ – meaning that plane spotting for this panellist, and whether he should go or not, was not a trivial or simple question. It was a moral question of human flourishing and its relation to other goods, rights, and responsibilities.

In the end, the panellist did go plane spotting, justifying the trip to Heathrow as the equivalent of travelling for swimming in his wife's case, in relation to rules at the time (‘I could do it under drive‐to‐exercise‐cum‐picnic‐sunbathe as per the regulations’), and on the basis of mitigation or hygiene measures he would take (‘all self‐contained with my sandwiches and bottle of juice’). With this particular dilemma resolved, however, the diarist just faced other dilemmas. What should he do about neighbours breaking the rules? Should he report them? How to balance being a good citizen (by helping to enforce the rules) against being a good neighbour (‘Don't want to cause aggro with our neighbours’). In the event, he sought advice from Facebook friends. Another dilemma set protecting the NHS against protecting the environment. ‘Trying to save on plastic’, he ‘picked some loose tomatoes’ at the supermarket. But then in order to weigh them, the cashier had to touch them. ‘It's a minefield’, wrote the panellist, before reporting on yet another dilemma. His daughter was ‘deeply concerned as we all are about the events in America following the murder of George Floyd. She was worried, conflicted and distressed in equal measure. Torn between wanting to join in the protests here but afraid of the impact of Coronavirus amongst the crowds’. How to balance staying home, managing risks to one's own health and responsibilities to the NHS and those it serves, against demonstrating support for the Black Lives Matter movement?

Many diarists found these dilemmas upsetting and exhausting. Having been given the responsibility for making decisions, they felt the burden of that responsibility and worried about making wrong decisions. Let us move to a different example. A company director in her 50s from Leeds (MT 2020 529) began her 12 May diary as follows: ‘Today my husband and I are arguing about whether he can play golf or not. The government advice is that they can play in pairs but he has also been told he is still shielding’. She continued:

He does not feel that going to play golf on his own and having no contact with anyone is going to increase his risk […] I want him to be able to enjoy his sport but I feel worried about the risks. It felt easier when no‐one could play golf or travel or work. Now there is so much to navigate and so much to decide. My mum is already talking about me visiting them again. But it is 200 miles by train to London […] and that exposes me to a whole lot of risk. ‘But what is our exit plan?’ my husband asks. I am somehow expected to know, to somehow be the grown up in all of this. He seems to expect me to set the rules for him and yet he does not really want that. I no longer know what to say to him about it all. ‘Yes it is unfair that you cannot go out’ and yes it is unfair that your asthma means you may not recover if you get the virus and yes I'd feel very guilty if I was to bring the virus home.

There is a lot going on here. Government rules and guidance were seen as unclear. Different parties in the argument began from different perceptions of risk. The diarist felt under pressure to somehow manage both risks (to herself on public transport) and responsibilities (to her mother, demanding a visit). She felt responsible for protecting her shielding husband and guilty for numerous things, from stopping him enjoying his sport in the present, to potentially bringing the virus home in the future. No wonder it ‘felt easier’ before 10 May when the pandemic was governed primarily through rules/morality, as opposed to freedom/ethics (which, for this diarist, still involved rules, but placed the burden for setting them – and the blame and guilt – on her).

In the last four paragraphs, we have seen that people often faced ethical dilemmas during the pandemic and found decisions hard to make. How, then, were these decisions ultimately made? One way, as we have seen, was through deliberation among family members (who ‘scratched their heads’ together), friends (e.g., on Facebook), and strangers (e.g., on internet forums). But another way, common to many writers for MO, was rules of thumb, or what behavioural scientists call ‘heuristics’: simplifying shortcuts allowing judgements in situations where actors lack information, experience, and feedback (Kahneman, 2012). Some of these rules of thumb encouraged pragmatic action over paralysis. We have to ‘muddle through’ (MT 2020 7), put our ‘best foot forward’ (MT 2020 14), ‘do our best’ (MT 2020 479), ‘just keep doing the next right thing’ (M5645). Some helped to justify greater risk‐taking. We should not ‘go overboard’ in taking precautions (M3462). ‘We have to crack on with life’ (K7050). After all, ‘life is for living’ (K7522). Alternatively, some heuristics helped to justify greater caution. We should ‘be on the safe side’ (D4736) and ‘play safe’ (W633). ‘One cannot be too careful’ and it is ‘better to be safe than sorry’ (L6048).

## 4 GEOGRAPHIES OF COVID‐19 AND OTHER EMERGENCIES

There are many ways for geographers to engage with the COVID‐19 pandemic. The disease and responses to the disease can be mapped. The (non‐)human causes of the pandemic can be studied. The uneven distribution of vulnerabilities and harms, especially across space and place, can be described and explained. The implications of the pandemic for globalisation, supply chains, borders, biopolitical surveillance, and cities can be studied. During 2020 and 2021, many such options were outlined in editorials in geography journals (e.g., Diaz & Mountz, 2020; Dodds et al., 2020; Sparke & Anguelov, 2020). During 2022, papers began to emerge taking up some of these options, not least in this journal (e.g., Herrick et al., 2022; Lin, 2022; Mould et al., 2022; Schliehe et al., 2022). In the present paper, we have touched on various geographical themes, including: rules governing geographical behaviour (e.g., ‘Stay Home’); coding of the home (as safe) and the outside world (as dangerous); technologies for protecting the home from the outside world (quarantine, sanitiser); and alternative imaginative geographies of the outside world as diverse (with more risky places, identified as crowded or dirty, and less risky places, identified as quiet and clean) or even the privileged location for certain goods (participation, sociality).

All such themes deserve more research. Our main concern in this paper, though, has been with good citizenship during the pandemic, especially when good citizenship is viewed through a geographical – contextual, situational, relational – lens. We agree with many psychologists that too much attention was given to non‐compliance during the pandemic. Most people complied with government rules – at least in the UK, at least during the early stages of the pandemic. However, we argue that too much attention was also given to compliance. The pandemic was governed by rules/morality, but also – especially during periods of lockdown relaxation – freedom/ethics (including common sense and personal responsibility). Governance through freedom/ethics begs a different set of questions to those focused on compliance.

We asked: what did people do when encouraged to use common sense and manage their own risk, responsibility, and geographical behaviour; and how did people experience all this? We found that responses varied by situation. Many people who saw themselves as vulnerable, or lived with people they saw as vulnerable, responded by taking extra care to minimise risks. Many key workers responded by taking extra care to leave the virus at work and not bring it home (so far as that was possible). Beyond this claim of variation by situation, however, five claims have been made about ‘diarists generally’ or ‘many panellists’. We now consider these claims in relation to available survey evidence, before clarifying the original contributions of the paper and the MO evidence on which it is based.

Diarists were generally confused by the shift in government messaging on 10 May from ‘Stay home, protect the NHS, save lives’ to ‘Stay alert, control the virus, save lives’. They viewed the new rules and guidance as less clear and more open to interpretation. How does this claim fit with relevant survey data from the period? The ONS Opinions and Lifestyle Survey (ONS, 2020) asked questions about ‘Coronavirus and the social impacts on Great Britain’ either side of 10 May (24 April–3 May and 14–17 May). A majority of people reported they had enough information to protect themselves from the virus, though this figure dropped from 88% to 80% over the period. A minority of people reported they had enough information about the UK's plan for dealing with the virus. Again, this figure dropped over the period (from 48% to 41%). Before 10 May, questions were asked about awareness of official government advice and stay home guidelines (a majority of people reported awareness), but these questions were not repeated after 10 May. Moreover, these questions about whether people had enough information to protect themselves or were aware of government advice do not really capture the same thing as confusion (on the part of citizens) or lack of clarity (on the part of government advice). One can have enough information to protect oneself – gathered from a variety of sources – or be aware of government advice, but think the messaging, rules, and guidance provided by government are confusing and lack clarity. A better set of survey questions for our purposes are those asked by King's College London and Ipsos MORI on 1–3 April (Policy Institute, 2020a) and 20–22 May (Policy Institute, 2020b). Between these two points, they found the proportion of people viewing the government response as confused and inconsistent rose from 42% to 59%, and the proportion of people viewing government communications and advice as helpful fell from 68% to 47%. The discontent expressed by many MO correspondents around the middle of May 2020 regarding messaging from the government and its general approach to the pandemic appears to have been the majority position in the UK.

A second claim we have made is that many panellists did not welcome the new freedoms announced on 10 May 2020. In these freedoms, they saw responsibilities. They wore the new demand to act responsibly like a heavy burden. Again, there are no survey questions from the time that precisely test our findings, but there are some relevant questions worth discussing. The ONS survey found strong support for the ‘Stay home’ approach prior to 10 May (96%), as did King's College London and Ipsos MORI polling (89%). After 10 May, the ONS did not ask about support for the new approach, but King's College London and Ipsos MORI did. Support fell from 89% for the ‘Stay home’ approach (1–3 April) to 38% for the relaxation of lockdown measures signalled by the ‘Stay alert’ approach (20–22 May). King's College London and Ipsos MORI polling in late May found that a majority thought the government was relaxing control measures too quickly (54%), a majority felt uncomfortable about sending their child to school (56%), and a significant minority felt uncomfortable about returning to the workplace (41%). The ONS survey also asked about feelings regarding the relaxation of lockdown. In mid‐May, 41% of respondents felt unsafe outside of their home (14–17 May). In late May, 63% felt unconfident about sending their children back to school in June (28–31 May). In early July, 70% felt uncomfortable about visiting the cinema and 60% felt uncomfortable about eating indoors at a restaurant (2–5 July). In mid‐May, a majority (65%) did say their lives were being affected by a lack of freedom and independence (14–17 May), but this is different from saying they wanted freedom and independence regardless of the costs. Overall, the survey data give an impression of a majority who supported the ‘Stay home’ approach and were not ready in May 2020, or even in subsequent months, for the relaxation of lockdown measures. Again, our evidence from MO is consistent with this overall picture.

A third claim made in previous sections is that many panellists sought to reduce risk to themselves and others by doing things like timing and locating their daily walks for quiet periods and places, and cleaning themselves, their clothes, and their homes more regularly. The ONS survey confirms that MO panellists were not unusual in doing such things during spring and summer 2020. Large majorities washed their hands more regularly (90%, 20–30 March), avoided physical contact with older and/or more vulnerable adults (91%, 24 April to 3 May), and avoided physical contact with others, staying 2 metres apart, when outside the home (93%, 24 April–3 May). King's College London and Ipsos MORI polling tells a similar story. Large majorities reported: washing their hands more regularly (93%, 1–3 April; 90%, 20–22 May); staying 2 metres apart from others when outside the home (94%, 1–3 April; 92%, 20–22 May); avoiding public transport (84%, 1–3 April; 82%, 20–22 May); avoiding places where many people gather (93%, 1–3 April); and using caution when handling deliveries and mail (73%, 1–3 April). A significant proportion of respondents reported washing or disinfecting items brought into the home (55%, 20–22 May) and changing or washing clothes on returning home (38%, 20–22 May).

We now move to the final two claims in this paper, which constitute its main contributions, not least because they are not well covered by available survey research. We have demonstrated that many MO correspondents, despite seeking to reduce risk to themselves and others where possible, took risks during spring and summer 2020 and justified that risk‐taking in multiple terms. Travel and visiting were justified if they allowed certain responsibilities to be fulfilled: caring responsibilities for others, but also for the self (mental health, in particular). They were also justified in terms of local infection rates (if comparatively low), options for mitigation (masks, gloves, sanitiser), practical considerations (regarding what is possible, given certain resources and opportunities), and feelings (regarding certain places perceived as safe/unsafe). Furthermore, we have demonstrated that many MO correspondents found themselves confronted by ethical dilemmas and required to balance multiple demands, rights, virtues, and goods. Those shielding at home worried about losing their independence. They also worried about how to participate and flourish. Those social distancing worried about how to reciprocate politely when invited into situations of social proximity. They also worried about how to care for others. Those using sanitiser, extra packaging, and personal protective equipment worried about their impact on the environment. For many people, such balancing acts were not easily completed. They used rules of thumb or heuristics to move forward beyond paralysis, but with little satisfaction regarding both process and outcome. In general, governance through personal responsibility was experienced by many as confusing, exhausting, a burden, an imposition.

We are aware of no survey evidence from the period that directly speaks to these findings on justifications and ethical dilemmas. Indeed, in common with qualitative data more generally, this is what MO materials have been traditionally good for: demonstrating not only people's preferences but also how people arrived at those preferences, and the extent to which they are ‘preferences’ at all. Regarding the COVID‐19 pandemic, MO materials are particularly well‐suited to demonstrating how good citizenship emerged from situations and involved: interpretation of rules and guidance; management of freedom, risks, and responsibilities; management of such items by way of deliberation and justification; and negotiation of ethical dilemmas. More broadly, MO materials are well suited to studying the ‘spaces of democracy’ (Barnett & Low, 2004) where actually existing, ordinary citizenships form through geographical action and awareness, but also argument, reasoning, interpretation, judgement, and justification.

Finally, as we have seen, experiences of managing risk and responsibility during the COVID‐19 pandemic were far from positive for many people. What, then, might be learned from this exercise – viewing good citizenship during the pandemic from a geographical perspective – for governing future emergencies? We finish with three lessons. First, when people act in emergencies, they do so in complex, ambiguous, diverse situations. Their actions are governed by rules, but also multiple and variable resources, opportunities, relations, commitments, and norms. We need a public conversation that recognises all this and takes us beyond the binary of compliance and non‐compliance. Second, to govern themselves in contexts of relative freedom, people need information, such as that provided to varying degrees of effectiveness by government, science, and media actors during the COVID‐19 pandemic. They also need practical support, such as that recommended by various commentators during the COVID‐19 pandemic (e.g., payments for those asked to self‐isolate by contact tracers). Then, crucially, they need justifications appropriate to the complex, ambiguous, diverse situations in which people find themselves. They need such justifications to replace the unsatisfactory rules of thumb currently relied on by many to navigate the ethical dilemmas and arguments about responsibility generated by emergencies when governed through freedom. Third, while focusing on what a better public conversation might sound like in future similar circumstances, we should also acknowledge that freedom and responsibility are not things people generally ask for during emergencies. In such times of complexity, uncertainty, and gravity, many people want government through rules/morality. They want the burden, blame, and guilt of deciding and acting to fall on experts and authorities. They wonder if government through freedom/ethics is appropriate to such events. Indeed, determining when and where governing through freedom is appropriate has become one of the central challenges for advanced liberal governmentality in an age of crises – financial, climate, public health, and geopolitical.

## FUNDING INFORMATION

British Academy [Grant number: COV19\200422].

## Supporting information


**Appendix S1**: The directives & The sample.

## Data Availability

Data supporting the results and analysis can be accessed at the Mass Observation Archive (see https://www.massobs.org.uk).
