# Supplementary material for: Beyond compliance: Good citizenship during the COVID‐19 pandemic
Source: Trans Inst Br Geogr. 2022 Nov 11:10.1111/tran.12587. Online ahead of print. doi: 10.1111/tran.12587 (PMC9874751; doi:10.1111/tran.12587)
Supplement: Supplementary file 1 — Appendix S1: The directives & The sample. [file TRAN-9999-0-s001.pdf]

SUPPLEMENTARY MATERIAL: BEYOND COMPLIANCE
Section 1: The directives

Special directive (sent to panellists on 17 March 2020)
Dear Mass Observers,<br><br>We hope you are all well at this difficult and ever-changing time.<br><br>We are writing to you following World Health Organisation declaration that COVID-19 / Coronavirus is now classified as a pandemic.<br><br>Yesterday, Boris Johnson warned Britons to avoid non-essential contact. Consequently, the MOA team are making plans to work from home. During this time we will continue making efforts to keep in touch with you all. However, this may mean that we are unable to acknowledge your Directive submission or enquiry. Please do continue to send in your contributions to the Archive and assume that it has arrived safely even if we don't acknowledge it.<br><br>We are currently finalising the Spring Directive so this will be with you in the next few weeks and hopefully this will be something nice to keep you focused during these strange times. In the meantime and over the coming months we would like you to please record your experiences, thoughts and opinions as the coronavirus unfolds. It is an unprecedented time and as with previous events in Mass Observation's history, we would like you to capture this in your writing. Some thoughts for you:<br><br>• Have you, or others you know, been effected by the virus? Have you been ill?<br>• Are you doing anything to protect yourself or others from the coronavirus? Have you self-isolated? Have you changed your behaviour?<br>• Have your shopping habits changed? Have you noticed any changes in the availability of food or other goods in the shops?<br>• What do you think about the UK government's response to the pandemic? We welcome thoughts about any other country's response to Covid-19.<br>• Where do you get your news about the virus? Have you noticed any jokes, memes or sources poking fun at the virus?<br><br>We wish you all good health. We are aware that for many of you the isolation will be very hard and will be thinking of you all.<br><br>Keep well and safe.<br><br>With best wishes,<br><br>Kirsty Pattrick (Mass Observation Projects Officer) and Jessica Scantlebury (Mass Observation Supervisor)
Spring directive (sent to panellists on 6 May 2020)
Part 1: Gender […]<br><br>Part 2: Covid19 (Update)<br><br>Please share your experiences and reflections on the challenging times we are currently experiencing. Below are some questions we would like you to think about.<br><br>Health. Please describe any physical or mental health issues you or anyone you live with have experienced during the lock-down period. Have you required any medical or professional help? If so, please tell us about this. How would describe your emotions in the last few weeks?

The Government. What are your thoughts on the political decisions that have been made? What steps do you think the British Government will make in the coming months?

News. Through what forms of media are you reading and/or hearing news of latest developments? Have you been watching the daily Government press broadcasts at 5pm? If yes, please tell us what your thoughts and experiences of this have been like. Have you or anyone you know experienced challenges with access to the news? There is a petition underway to the UK Government and Parliament to include a British Sign Language Interpreter in these briefings. BSL interpreters are used for Nicola Sturgeon's briefings in Scotland. What do you think about this?

Work and home life. Have you been working during the lockdown? Or maybe you have been furloughed? Please share your experiences. How has lockdown impacted your home life? Have you been home schooling children? Has it affected any relationships with people you live with? How have you managed these challenges?

Technology. Many people have been learning to use new forms of technology during the crisis. What has your experience been of this? Have you been keeping in touch with friends, family and/or work colleagues with tools such as FaceTime, Zoom, Houseparty or WhatsApp? If so, what has this been like? Have you re-connected with people or have you found this a challenge or a pressure? Have you been using social media platforms such as Facebook, Twitter and Instagram to keep up to date or escape from the events?

Shopping and food. What have been your experiences of buying groceries either in shops or online? Have you experienced any challenges? Have your eating/cooking habits changed or stayed the same?

Entertainment and Leisure. We would like you to share your experiences of how you have been entertaining yourselves. Have you taken up any hobbies, learnt new skills or games, or increased your exercise? Maybe you have completed some DIY jobs? We would also like to hear what you have been watching, listening to and/or reading.

Personal hygiene. We are interested to know whether your habits of personal hygiene have changed in recent months. Has the regularity of your washing, shaving, personal care been any different since the period of lock-down? If so in what way and how do you feel about this?

Sleep. There have been many reports of people experiencing insomnia and nightmares in recent weeks. We would like to hear about your sleep and those of your family or people that you live with. If you have experienced difficulties, what have you been doing to help relieve this? Have you had any interesting dreams? Please describe.

Call for 12th May day diaries (circulated widely in the weeks prior to 12 May 2020)

In 2020 the Mass Observation Archive will be repeating its annual call for day diaries, capturing the everyday lives of people across the UK. The written diaries will be stored in the Archive at The Keep and be used by a wide range of people for research, teaching and learning. This is the 10th Anniversary of the 12th May Diary project.

In 1937 Mass Observation called for people from all parts of the UK to record everything they did from when they woke up in the morning to when they went to sleep at night on 12th May. This was the day of George VI's Coronation. The resulting diaries provide a wonderful glimpse into the everyday lives of people across Britain and have become an invaluable national resource for those researching countless aspects of the era.

As we post this call, the UK is in lockdown because of the COVID-19 pandemic. We don't know how life will be on the 12th May, but we would like your help to document it. Please tell your family and friends. It will be valuable to have a collection from people of all ages across the UK.

As it is the 10th anniversary of the modern 12th May diary call (first issued in 2010), we would like you to reflect on the last 10 years. How has your life changed?

Ideally, diaries should be in electronic form as email attachments (Word documents preferably) and sent to moa@sussex.ac.uk We will accept physical/hard-copy diaries but advise that these are posted after the period of lockdown.

Schools and Community Groups:

We really welcome diaries from school children, community groups, or other organisations. This may be an activity for children and families during this time of home working and home schooling. Diaries can be written in any style and can include drawings.

Packs on our website are designed for schools and groups wanting to post their diaries to the Archive. Individuals can also use the diary template to submit their entry if they wish but please complete only the first page.

Summer directive (sent to panellists on 7 August 2020)

Part 1: Covid-19 and Time

COVID-19 has disrupted life as we know it. For many of us, our daily rhythms and routines have altered significantly following different government and workplace guidelines. For some, the days may seem to have bled into each other while for others the pace of life has become more frenetic. Time is generally something that is taken for granted but the pandemic has made many of us notice it in new ways. In this Directive, we ask you to tell us how time in your daily lives may have changed – or stayed the same – as a result of the pandemic. At this moment (August 2020), as lockdown is gradually easing in the UK, we would like you to please look back on your experiences of time and COVID-19 so far, consider what it means to you for life to get 'back to normal' (if it is) in the present day, and imagine what you think the future might hold.

As always, please include anything that is important to you that you feel is missing from our questions.

Rhythm and routine of the day. In what ways, if any, has 'time' in your daily life been disrupted?

In the first days of the lockdown, did your daily rhythms, routines, or habits change? If so, please say how and describe how this felt. Can you recall how these changes came about? Were they connected to specific government regulation or advice?

In what ways have different aspects of your life sped up or slowed down? For example, have you been furloughed from work or taken on caring responsibilities? Maybe you've been studying online or have taken up new hobbies. Please describe your experience of this.

Many people have spent more time than usual in their local environments. If this is the case for you, have you noticed anything in particular about the rhythms of the environment around you, such as the timing of seasons, plants, animals, the sun and the moon?

Are your rhythms and routines changing again now? If so, in what ways? Do you anticipate them changing again in the future? If so, please describe how you feel about this?

Home life. For those of you spending most of your time at home, how do you make distinctions between different times of the day? Do you describe these times – to yourself or members of your household – in any new ways since lockdown? For example, do you have particular times of the day you leave the house to take a walk or have meals? What factors influence the organisation of your time?

For those of you living in households with others, how do you negotiate time in relation to one another? Do some people's time matter more than others? Is this determined by work, caring responsibilities or maybe access to computers?

In what ways have your lives come to be 'in synch' with or at odds with one another over the period of lockdown?

What changes do you anticipate in the time of your home life in the future?

Media and technology. Have media and technologies featured in any daily life changes – for example, speaking to family or colleagues using online tools, having online medical appointments and/or shopping online? If so, has using these tools made a difference to how you manage, schedule or plan your time?

Did other materials – for example, notepads, diaries, wall calendars – feature as well? What did you use them for?

If you used new media, technologies or materials, or changed how you used them as a result of COVID-19, do you anticipate that you will keep using them in the same way in the future? The Spring Directive included a similar question. If you answered it, have your views changed?

Waiting. For some people, it seems as if life has been put 'on hold' during the pandemic. Does this feel relevant to you? If so, please say how. Have you experienced a particular pause or a delay, for instance in waiting for appointments, activities, a new job or for work to resume?

What is your experience of waiting? For example, you may have spent time queuing outside shops, waiting for deliveries, or waiting to see friends or family. Please describe how these times felt.

For those who have been directly or indirectly affected by illness during the pandemic, how have you experienced time and waiting in particular in relation to incapacity, recovery, loss and grief?

Part 2: Black Lives Matter […]

Section 2: The sample

Gender

	Male	Female	Other	Total
12 May diaries	23	37	0	60
Special and Spring 2020	25	35	0	60
Summer 2020	25	35	0	60

Notes: The 12 May diaries were sampled from the first 500 catalogued by the Archive. Responses to the Special and Spring directives were archived together. We sampled the same 60 panellists from the Special and Spring collection and the Summer collection.

Age

	18-29	30-39	40-49	50-59	60-69	70-79	80-89	90-99	Total
12 May diaries	8	12	11	9	9	7	3	1	60
Special and Spring 2020	3	7	7	6	11	18	6	2	60
Summer 2020	3	7	7	6	11	18	6	2	60

Country/region of the UK

	England									Wales	Scotland	N Ireland	Total
	E Mids	E of England	London	NE	NW	SE	SW	W Mids	Yorks and Humber				
12 May diaries	3	5	12	2	4	12	9	3	4	3	3	0	60
Special and Spring 2020	5	4	6	3	6	14	5	4	5	6	2	0	60
Summer 2020	5	4	6	3	6	14	5	4	5	6	2	0	60

Occupation

	Managers, directors, senior officials	Professional	Associate professional and technical	Administrative and secretarial	Skilled trades	Caring, leisure, and other service	Sales and customer service	Process, plant, and machine operatives	Elementary	Other	Total
12 May diaries	7	23	6	7	3	0	4	0	0	10	60
Special and Spring 2020	6	12	16	8	3	4	2	1	0	8	60
Summer 2020	6	12	16	8	3	4	2	1	0	0	60

Note: ‘Other’ includes student, unemployed, self-employed, and retired (all where no further information available e.g. sector or previous occupation).
